# Environmental Response of 2D Thermal Cloak under Dynamic External Temperature Field

**DOI:** 10.3390/e22040461

**Published:** 2020-04-18

**Authors:** Yiyi Li, Haochun Zhang, Mingyuan Sun, Zhenhuan Zhang, Haiming Zhang

**Affiliations:** School of Energy Science and Engineering, Harbin Institute of Technology, Harbin 150001, China; 18b902056@stu.hit.edu.cn (Y.L.); 19S002057@stu.hit.edu.cn (M.S.); 19S002033@stu.hit.edu.cn (Z.Z.); 19S102083@stu.hit.edu.cn (H.Z.)

**Keywords:** thermal cloak, dynamic environment, local entropy production rate, interaction effect

## Abstract

As a typical representative of transformation thermodynamics, which is the counterpart of transformation optics, the thermal cloak has been explored extensively while most current research focuses on the structural design instead of adaptability and practicability in a dynamic environment. The evaluation of energy processes involved in the thermal cloak under dynamic conditions are also lacking, which is essential to the engineering application of this functional structure. In this paper, based on the dynamic environment of a sinusoidal form with ambient amplitude, distribution density, phase, and temperature difference as variables, we evaluated the cloaking performance and environmental response of a 2D thermal cloak. Considering the heat dissipation and energy loss in the whole procedure, local entropy production rate and response entropy were introduced to analyze the different influences of each environmental parameter on the cloaking system. Moreover, we constructed a series of comprehensive schemes to obtain the fitting equation as well as an appropriate scope to apply the thermal cloak. The results are beneficial to the novel use of the concept of entropy and valuable for further improving the working efficiency and potential engineering applications of the thermal cloak.

## 1. Introduction

As an efficient tool to design novel functional elements through curvilinear coordinate transformations, transformation optics (TO) theory, originally developed from the electromagnetic field [1,2], enables steering of the electromagnetic wave with precise control of points in space and their refractive index, which can be achieved by mapping the curved space onto a distribution of metamaterial. Considering that the form invariance of governing equations under coordinate transformations is the essence of such a process, this basic theory has been applied to other physical fields, including optics [3], acoustics [4], and elastodynamics [5], realizing various regulatory structures represented by the invisibility cloak, which can hide targets without disturbing external fields. After the invariability of the heat conduction equation is demonstrated, characteristic heat phenomenon analogues to the cloaking effect in other physical fields have been widely explored in the diffusion field using an invisibility structure, namely the thermal cloak [6]. Through placing a qualified thermal cloak outside the target object in the thermal field, heat flows that would have struck the object target will be reflected and guided around the target object, then back to the original propagation path. Hence, the target object can be concealed and almost no disturbance appears in the external thermal field, making the observers unaware of these arrangements. Metamaterial was employed [7] to explore a bifunctional cloak based on the efficient medium theory. The strict requirement of extreme anisotropy was broken [8] by uniformly superposing concentric homogeneous materials with different thermal conductivities. A Jacobi matrix was also applied to design a 2D thermal cloak and extend TO theory to transformation thermodynamics [9]. According to the emerging theoretical design of thermal cloaks with different shapes and dimensions, relevant simulations and experiments have been carried out to explore the practical applications. Through alternately filling copper and polydimethylsiloxane (PDMS) in the cloaking region [10], the temperature gradient distribution in heat conduction procedure was experimentally observed for the first time. Making use of copper with a thickness of 100 μm, a 3D ultra-thin thermal cloak was prepared with metal processing technology [11]. In order to improve the maneuverability, polystyrene and alloy were picked as internal and external material, respectively, to design a new type of bilayer thermal cloak [12]. 

These simulated and experimental evidences can intuitively show the function of the thermal cloak. However, in addition to the characteristics of the structure itself, very few studies exist pertaining to the relationship between the cloaking effect and external environment. To simplify formula derivations and theoretical calculations, related ambient conditions were usually defined as fixed values. This is just a rare case in actual situations and dynamic environmental conditions with uneven distributions are more common. Thus, it is very necessary to explore the response of the thermal cloak in different environmental conditions and to evaluate the corresponding effect of the structure with an appropriate index, which is valuable and instructive for the future optimization and development of this typical device. 

Though qualitative indexes such as temperature deformation (TD) have been used in previous studies, research on the thermodynamic performance of the system consisting of thermal cloak and external environment is insufficient. Although equilibrium and stability were emphasized in classical science, now, irreversible processes can be observed in diverse fields from chemistry and biology to cosmology. These irreversible phenomena can be directly characterized through the concept of entropy, so a clear explanation of the relation between the entropy production as well as its rate and irreversible processes is necessary [13]. For a colloidal particle and general stochastic dynamics following a master equation, entropy production along a single trajectory can be obtained by adding entropy production of the system and of the medium. Regardless of time-dependent driving, initial conditions, and length of trajectories, this total value always obeys an integral fluctuation theorem [14]. Exact formulas of the entropy production rate in systems controlled by linear Langevin equations were also derived [15]. In order to correctly reflect the physical assumptions used for the current estimation of essential physics, maximum entropy production (MEP) was suggested to be equivalent to maximum entropy (MaxEnt) inference algorithm that translates physical assumptions into macroscopic predictions and can be applied to non-equilibrium systems rather than physical principles [16]. To characterize self-organized structures, the “minimum entropy production rate (EPR) principle” is usually utilized, and sometimes conflicts with “maximum EPR states”. Thus, a dual relation between the minimum and maximum principles was illustrated [17]. Further, thermodynamic characteristics including heat flow and local entropy production have been widely studied in bio-systems. A hypothesis was proposed and experimentally validated whereby steady states, which are actually not stable and only exist in characteristic time, are contained in the hierarchy of living systems. Considering that the long-term biological evolution should lead to the formation of living systems with minimal possible deviation from steady states, a difference between the current value of the entropy production and the entropy production value in the closest steady state is small enough to satisfy the laws of linear thermodynamics, which can potentially describe living systems [18]. As open thermodynamic systems, cells as well as their biological behaviors were explored through analyzing irreversible wasted heat generated inside the system and related to external perturbations [19,20]. To systematically describe the entropy production rate associated to irreversible processes, a theoretical model was established to calculate the entropy production rate in the minimum living system and applied to the case of glucose catabolism in normal and cancer cells. The results are beneficial to a better understanding of the self-renewal and physiopathologic process, and provide support for cancer detection [21]. For biological metabolic pathways, including lactic acid fermentation and respiration, the rate of entropy in a cell involving fermentation and respiration processes in the glucose catabolism of living systems was calculated [22], and an analogy was made between mechanics and thermodynamics by defining and expressing the entropy density acceleration with the time derivative of the entropy density rate to study the heat and mass transfer during glucose catabolism in living systems and their relation with entropy production [23]. In practical engineering applications, almost all heat transfer processes are irreversible, and the consequent heat dissipation cannot be ignored. 

Owing to the flow of heat, entropy production as well as its rate will appear as agents of energy disorder and vanish if the system reaches equilibrium [13]. Considering this, the entropy production rate is an effective estimation, and not only indicates the energy loss directly, but can help to minimize the loss and make the process closer to an ideal situation [24,25,26,27,28]. Therefore, in this paper, we established a 2D thermal cloak based on previous work [10] and combined a dynamic temperature field in the sinusoidal form. The variation of the local entropy production rate [29,30] of the structure with ambient amplitude, distribution density, phase, and temperature difference were calculated. Moreover, we introduced the concept of response entropy [29] to better reflect the cloaking performance by integrating the irreversible energy loss in the functional region and its surroundings. Furthermore, inspired by extensive manufacturing fields [31], response surface methodology (RSM) was selected to explore the interactions among different environmental variables, from which we can evaluate the environmental response and determine the suitable applications of the thermal cloak.

## 2. Theoretical Method and Establishment of Models 

### 2.1. TO-Based Theoretical Method

**Figure 1:**
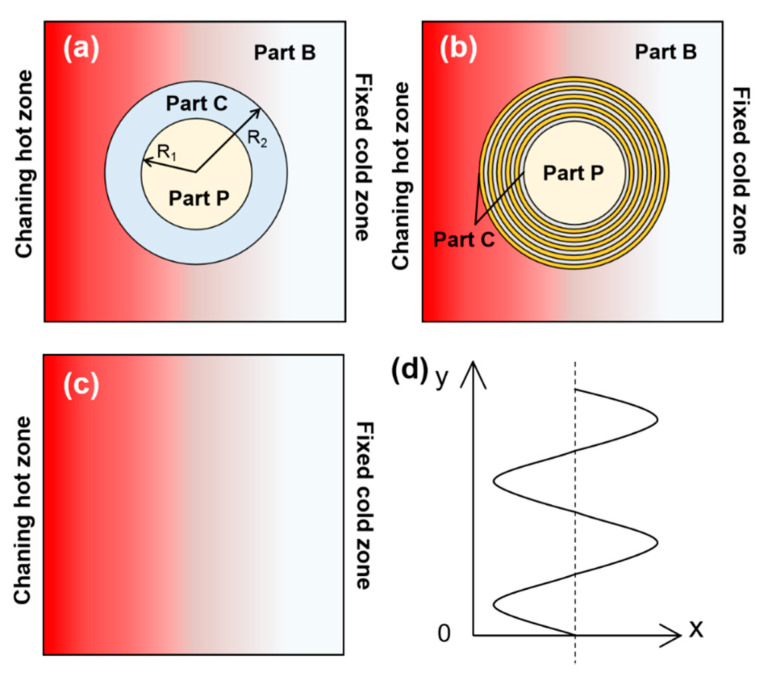
Schematic of thermal cloak, (**a**) diagrammatic sketch, (**b**) annulus structure with 10 composite layers, (**c**) Bare plate structure, (**d**) *T*_H_ of sinusoidal distribution under the control equation *T*_H_ = *Z*sin[*w*(*k*π/*w* + *t*/*w* + *ϕ*/*w*)] + *T*_ave_, where *Z* denotes amplitude, *w* is frequency with fixed value *w* = 0.05 rad/s, *k* is distribution density representing concentration of temperature change along the y axis (the wall direction), *t* is time, *ϕ* is phase and *T*_ave_ denotes average hot end temperature.

Referring to the space mapping and coordinate transformations of previous work, we need to clarify the heat conduction mechanism in the distorted polar coordinates. The two-dimensional heat conduction equation of the cloaking structure in the transformation space can be expressed as [29]:

(1) $$\rho c\frac{\partial T}{\partial t}=\frac{1}{r}\frac{\partial }{\partial r}\left(\kappa_{r}r\frac{\partial T}{\partial r}\right)+\frac{1}{r^{2}}\frac{\partial }{\partial \theta }\left(\kappa_{\theta }\frac{\partial T}{\partial \theta }\right)$$

where *ρ* and *c* respectively denotes density (kg·m^−3^) and specific heat capacity (J·kg^−1^·K^−1^) in the transformed space, while *κ_r_* and *κ_θ_* are the thermal conductivity components (W·m^−1^·K^−1^) in radius and azimuth directions of the cloaking region. Then, we adopt the specific expression of thermal conductivity in radius and azimuth directions of the functional part with radius interval *R*_1_ < *r* < *R*_2_ (see Figure 1a) as follows [9,10]:

(2) $$\kappa_{r}=\kappa_{0}{\left(\frac{R_{2}}{R_{2}-R_{1}}\right)}^{2}{\left(\frac{r-R_{1}}{r}\right)}^{2}\leq \kappa_{0}$$

(3) $$\kappa_{\varphi }=\kappa_{0}{\left(\frac{R_{2}}{R_{2}-R_{1}}\right)}^{2}\geq \kappa_{0}$$

where *κ*_0_ is the thermal conductivity of background domain. According to Fourier’s law, the heat flow distribution in the functional region corresponding to the above thermal conductivities can be obtained:

(4) $$q_{r}=-\kappa_{0}{\left(\frac{R_{2}}{R_{2}-R_{1}}\right)}^{2}{\left(\frac{r-R_{1}}{r}\right)}^{2}\frac{\partial T}{\partial r}$$

(5) $$q_{\theta }=-\kappa_{0}{\left(\frac{R_{2}}{R_{2}-R_{1}}\right)}^{2}\frac{1}{r}\frac{\partial T}{\partial \theta }$$

Concentrated on thermodynamic performance of the system consisting of thermal cloak and external environment, the concept of local entropy production rate *S*’_*gen*_ (W·K^−1^·m^−2^) [27,29,30] and response entropy *S*_*re*_ [29] are introduced to describe the cloaking characteristics:

(6) $$\begin{array}{ll} S_{gen}^{\prime } & =\frac{1}{r}\frac{\partial }{\partial r}\left(\frac{q_{r}}{T}\right)+\frac{1}{r^{2}}\frac{\partial }{\partial \theta }\left(\frac{q_{\theta }}{T}\right)-\frac{1}{T}\dot{q}\\ & =\frac{1}{r}\frac{\partial }{\partial r}\left[\frac{1}{T}\left(-\kappa_{r}\frac{\partial T}{\partial r}r\right)\right]+\frac{1}{r^{2}}\frac{\partial }{\partial \theta }\left[\frac{1}{T}\left(-\kappa_{\theta }\frac{\partial T}{\partial \theta }\right)\right]-\frac{1}{T}\dot{q}=\frac{\kappa_{r}}{T^{2}}{\left(\frac{\partial T}{\partial r}\right)}^{2}+\frac{\kappa_{\theta }}{r^{2}T^{2}}{\left(\frac{\partial T}{\partial \theta }\right)}^{2}\\ & =\frac{\kappa_{0}}{T^{2}}{\left(\frac{R_{2}}{R_{2}-R_{1}}\right)}^{2}{\left(\frac{r-R_{1}}{r}\right)}^{2}{\left(\frac{\partial T}{\partial r}\right)}^{2}+\frac{\kappa_{0}}{r^{2}T^{2}}{\left(\frac{R_{2}}{R_{2}-R_{1}}\right)}^{2}{\left(\frac{\partial T}{\partial \theta }\right)}^{2} \end{array}$$

(7) $$S_{re}=\left|\frac{\iint_{A_{c,C}}S_{gen}^{\prime }dA-\iint_{A_{p,C}}S_{gen}^{\prime }dA}{\iint_{A_{c,B}}S_{gen}^{\prime }dA-\iint_{A_{p,B}}S_{gen}^{\prime }dA}\right|$$

According to *S*′_*gen*_ that changed with structural parameters and temperature gradient at different locations, the energy randomness and irreversible loss of the system at different times under sinusoidal temperature fields varying with the ambient amplitude, distribution density, phase, and temperature difference can be evaluated. In Equation (7), the subscript of area parameter *A* includes two parts, the first letter (*c* or *p*) denotes model type (cloaking structure and bare plate), the latter one denotes concrete calculating area, as shown in Figure 1a. The key measure to improve the working efficiency of the thermal cloak is to reduce the disturbance to an external thermal field and increase the irreversible loss in the cloaking region when the irreversible loss in the central protected region is small enough. A larger *S*_*re*_ means the cloaking system contains the most heat dissipation in the cloaking area and less entropy production in the protected area, leading to a better cloaking performance. Hence, the value of *S*_*re*_ is regarded as the integration of entropy production between a functional region and its surroundings, which is a productive evaluation index of cloaking performance and environmental response. 

### 2.2. Establishment Process of Models

In Figure 1b, we established the 2D thermal cloak model with anisotropic thermal conductivities in the functional region by neglecting the thickness of the system and considering that the effect of heat convection can be decreased with a vacuum chamber [32]. The whole block was divided into three parts: the background part (Part B), the cloaking part (Part C), and the protected part (Part P). The background was a square plate with side length of 200 × 200 mm. The left side as hot end *T*_H_ owned the temperature of sinusoidal distribution as Figure 1d and constant temperature *T*_L_ = 293 K on the right side as cold end. Part C was a composite structure, alternately filled with copper and PDMS. The two materials were placed layer by layer in the form of 10 annulus, of which the thickness is 2 mm each. The efficient thermal conductivity of this alternate composition has the following expression [10]:

(8) $$\kappa_{eff}=\overset{10}{\underset{i=1}{\sum }}\frac{A_{i}}{A_{C}}\left(f_{i}\kappa_{cu}+\left(1-f_{i}\right)\kappa_{PDMS}\right)$$

where *i* represents layer number and *f*_i_ represents area fraction of copper. *A*_i_ denotes the area of each annulus and *A*_c_ denotes the area of the whole Part C, that is, *A*_i_/*A*_c_ represents the area component of relative annulus region. Enlightened by previous work [10], we determined the thermal conductivity of each material layer as *κ*_1_ = 0.15, *κ*_2_ = 390, *κ*_3_ = 2.63, *κ*_4_ = 385, *κ*_5_ = 9.85, *κ*_6_ = 385, *κ*_7_ = 18.5, *κ*_8_ = 375, *κ*_9_ = 28.5, *κ*_10_ = 370 from the inside to the outside respectively. Thus, we can calculate the efficient thermal conductivity of the cloaking region *κ*_eff_ = 206 from the equation above. We set the mixture of copper and PDMS as the background material with thermal conductivity *κ*_0_ = 88 to provide about 30% local effective specific heat variations and a round copper sheet with radius of 30 mm was put in Part P as the protected object. In addition, we established a bare plate model for comparison in Figure 1c with the same boundary conditions and background materials as the cloaking model. The heat transfer process and thermodynamic characteristics in the computational domain were simulated by ANSYS Fluent.

## 3. Entropy Analysis of Performance of Thermal Cloak 

### 3.1. Temperature and Local Entropy Rate Distribution of Thermal Cloak

**Figure 2:**
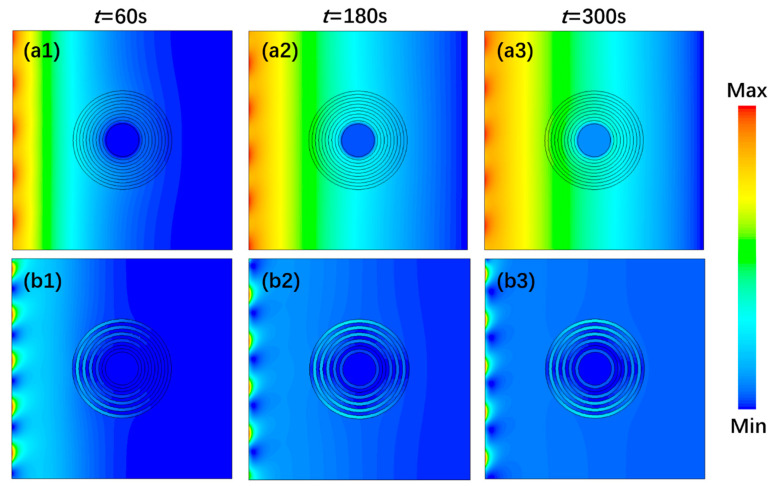
Distribution of temperature and local entropy production rate of the cloaking system at different times, (**a1**–**a3**) temperature distribution (**b1**–**b3**) local entropy production rate.

The distribution of temperature and local entropy production rate *S*’_*gen*_ of the cloaking system at times 60 s, 180 s, and 300 s are shown in Figure 2. The temperature and *S*’_*gen*_ in Part P remained extremely low, indicating a conspicuous cloaking performance that not affected by external temperature changes. At the initial stage of the heat conduction process, *S*’_*gen*_ was much higher near the hot end side and accumulated in a certain degree at the left outer boundary of the annulus structure. With increasing time, *S*’_*gen*_ encircled Part C as the heat flow dodged around the cloaking structure and transferred to both sides along the structure contour. Due to the energy randomness caused by alternating thermal conductivities in composite layers, the value of *S*’_*gen*_ in odd layers was quite different from that in even layers, and the highest value appeared in the upper and lower area of the outermost material ring, resulting in quite a lot heat dissipation and an irreversible loss of energy. What’s more, because the whole energy distribution tended to be uniform, the central temperature rose slightly over time and *S*’_*gen*_ in Part B with its boundary accumulation decreased gradually, indicating the system will eventually reach an equilibrium.

**Figure 3:**
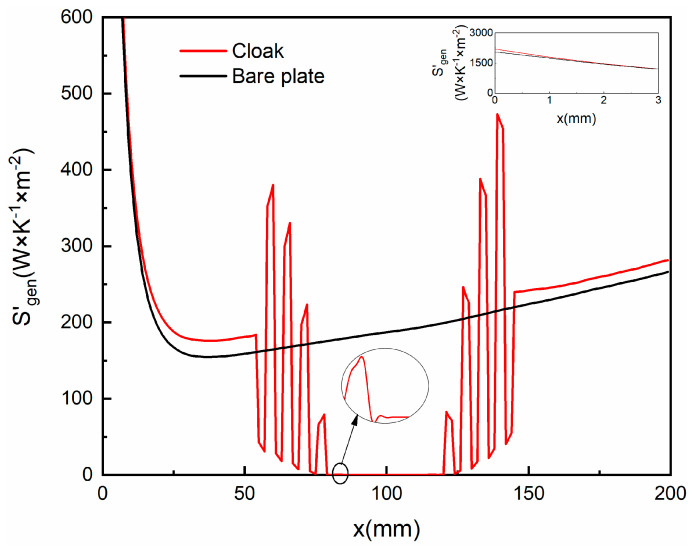
Local entropy production rate on symmetry line (*y* = 100 mm) at *t* = 300 s.

In Figure 3, we extracted value of *S*’_*gen*_ at time *t* = 300 s on the symmetry line (*y* = 100 mm) to make a quantitative comparison. The effectiveness of the cloaking structure was clearly validated not just because *S*’_*gen*_ in Part P was low enough in all situations but the changing trend in Part B kept consistent both in cloaking model and bare plate model, numerically closed either. Also, there existed distinct fluctuations of *S*’_*gen*_ in Part C resulting from the numerous differences of thermal conductivities between adjacent layers, meaning that heat dissipation and irreversible energy loss of the system was mostly contributed from the composite region. Relatively small thermal conductivity of PDMS in odd layers owned lower heat propagation speed and temperature gradient, leading to more energy randomness that contrary to the situation of copper in even layers.

**Figure 4:**
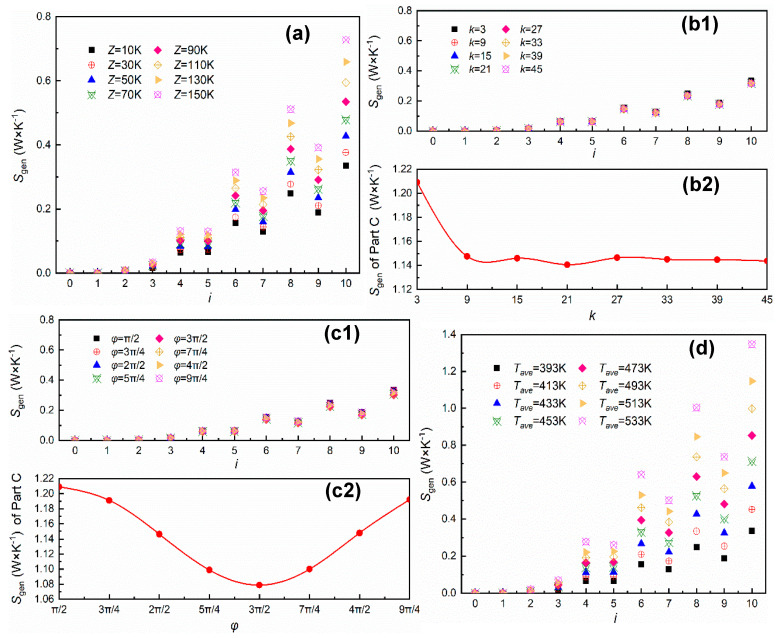
The effect of each environmental parameter on cloak structure, (**a**) layer-*S*_*gen*_ changes with ambient amplitude *Z*, (**b1**) layer-*S*_*gen*_ changes with distribution density *k*, (**b2**) *S*_*gen*_ of Part C changes with distribution density *k*, (**c1**) layer-*S*_*gen*_ changes with phase *ϕ*, (**c2**) *S*_*gen*_ of Part C changes with phase *ϕ*, (**d**) layer-*S*_*gen*_ changes with temperature difference (adjusted by *T*_ave_).

In order to specify the influence mechanism of the dynamic temperature field on cloaking performance, we arranged a series of simulation schemes with ambient amplitude, distribution density, phase, and temperature difference as variables. Since the temperature gradient distribution of the system differed in each environmental condition, it is more intuitive to take the integration of local entropy production rate as analysis standard:

(9) $$S_{gen,i}=\iint S_{gen}^{\prime }dA_{i},\;i=0,1,2,\ldots ,10$$

where *i* = 0 represents Part P and other numbers refer to layer number. Figure 4 reflects the effect of different environmental parameters on the thermodynamic state of the system. Overall, the *S*_*gen*_ distribution demonstrated inter-layer differences consistent with the distribution of *S*’_*gen*_, and value of *S*_*gen*_ at *i* = 0 tended to be infinitesimal in all conditions, implying that all schemes were at work. An uneven external environment with different degrees of thermal disturbance in the system required a certain heat dissipation capacity of the thermal cloak to better realize the protecting function. In Figure 4a, *S*_*gen*_ showed an obvious increasing trend with the increase of ambient amplitude *Z*, indicating an improving ability of environment-adapted heat dissipation. When distribution density *k* and phase *ϕ* varied in the given range in Figure 4b1,c1, changes of *S*_*gen*_ between layers were closed and difficult to observe directly, so we calculated *S*_*gen*_ of whole Part C under different variable values, as Figure 4b2,c2 show. With the increasing *k*, *S*_*gen*_ decreased first, then floated down the horizontal line with *S*_*gen*_ = 1.4, and *S*_*gen*_ first decreased, then increased as *ϕ* increased, which reached minimum valued at *ϕ* = 3π/2. In Figure 4d, with the increase of *T*_ave_, *S*_*gen*_ was monotonically increased, showing the scheme under environment with larger temperature difference exhibited better heat dissipation ability.

**Figure 5:**
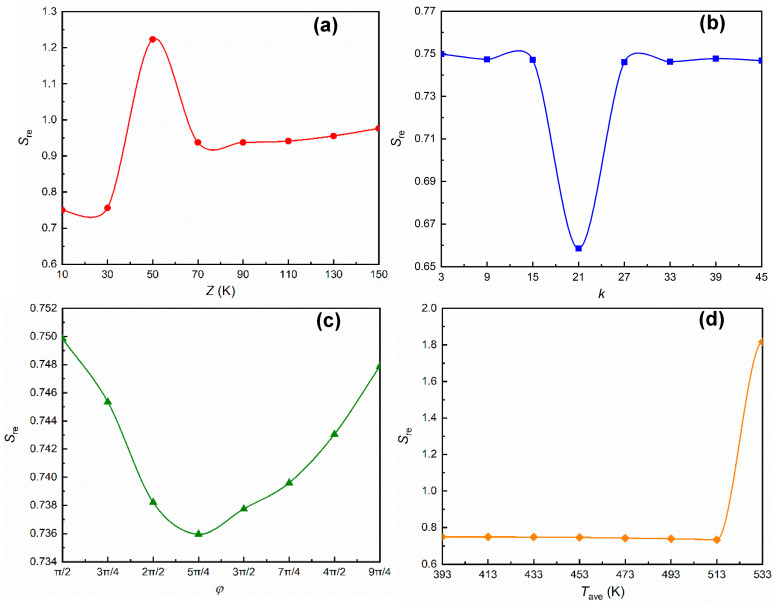
The effect of each environmental parameter on *S*_*re*_, (**a**) changes with ambient amplitude *Z*, (**b**) changes with distribution density *k*, (**c**) changes with phase *ϕ*, (**d**) changes with temperature difference (adjusted by *T*_ave_).

What’s more, considering that the cloaking performance is the product of strong heat dissipation ability in multi-layers and small thermal disturbance on the protected area, we extracted the value of response entropy *S*_*re*_ in Figure 5 to give a more comprehensive description about cloaking characteristics. As shown in Figure 5a,b, when *Z* and *k* changed in the given range, *S*_*re*_ had the highest and the lowest peaks, respectively, and the change was relatively stable around the peak, indicating that there existed a specific scope for the cloak application. In Figure 5c, *S*_*re*_ first increased, then decreased with the increase of *ϕ*, which was similar to the changing trend of *S*_*gen*_. As *T*_ave_ increased, *S*_*re*_ basically changed along a horizontal straight line, but jumped to twice the line value when *T*_ave_ exceeded 513 K, as Figure 5d shows.

### 3.2. Comprehensive Evaluation of Cloaking Performance

From the above discussions, the cloaking performance of the multi-layer structure was not consistent for each environmental parameter, and we were unable to directly evaluate the cloaking effect under comprehensive external conditions. Thus, based on the change of *S*_*re*_, the RSM method [31] was introduced to evaluate interaction effects among environmental variables. In Table 1, we set the variation range of ambient amplitude *A* and distribution density *k* to 10–150 K and 3–45, respectively. The phase *ϕ* changed within π/2–9π/4, temperature difference was adjusted by *T*_ave_ which varied in the range of 393–533 K. For convenience, the four environmental variables were symbolled by E, F, G, and H. Through arranging different environmental variables, 29 simulation schemes were conducted and the modified cubic model results of the analysis of variance (ANOVA) are presented in Table 2. Values of “R-Squared”, “Adi R-Squared”, and “Adeq precision” indicate an adequate signal to noise ratio, showing that the model can reasonably navigate the comprehensive design space and identify the interaction effect [33]. Therefore, we can obtain the following fitting equation:

(10) $$\begin{array}{c} S_{re}=0.97-0.14E-0.073F-0.051H+0.13EF-0.29EH-0.26FH-0.029F^{2}\\ +0{.12H}^{2}+0.2E^{2}F+0.29E^{2}H+0.26EF^{2}+0.4EG^{2}+0.25F^{2}H+0.094FG^{2} \end{array}$$

**Figure 6:**
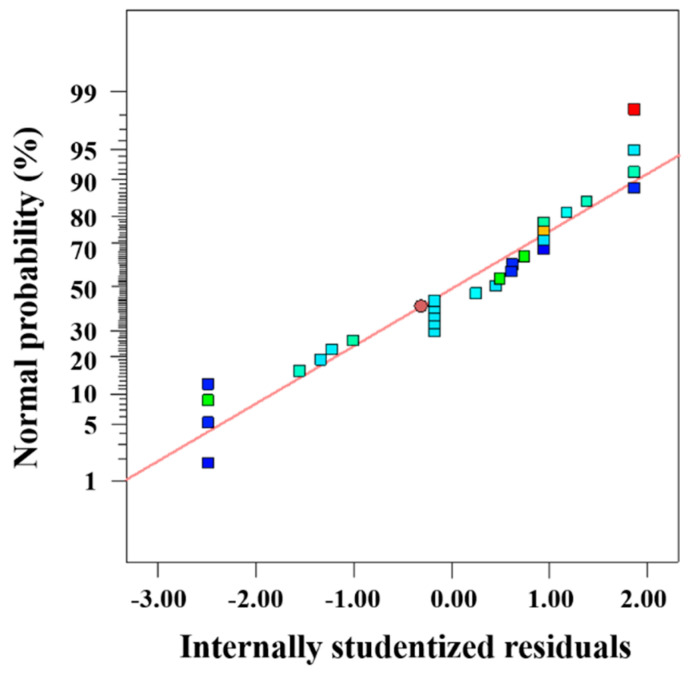
Normal plot of internally studentized residuals.

Figure 6 illustrates the normal plot of residuals for *S*_*re*_, the internally studentized residuals basically concentrated on the diagonal line, further indicated the validity and reference of the fitting equation. The fitting equation can be analyzed according to the F value and *p* value of each item that represented statistical significance. Generally, a higher F value and a lower *p* value denote larger contribution in the fitting equation and the item would be considered significant if its *p* is value no more than 0.01 [33]. Clearly, most of the items have a great impact on *S*_*re*_ of the system, while minority coded parameters including average hot end temperature *T*_ave_ (H), squared distribution density (F^2^), as well as the combination of distribution density and phase (FG^2^) played a relatively small role. Moreover, the items consisting of single parameter involved in the fitting equation were all negatively correlated with *S*_*re*_ while different combinations of these parameters were likely to have different effects, thus it was necessary to further explored these interaction relationships. Based on the significance of interaction items shown in Table 2, we performed a related 2D contour map to analyze the interaction regulations between any two environmental variables.

**Figure 7:**
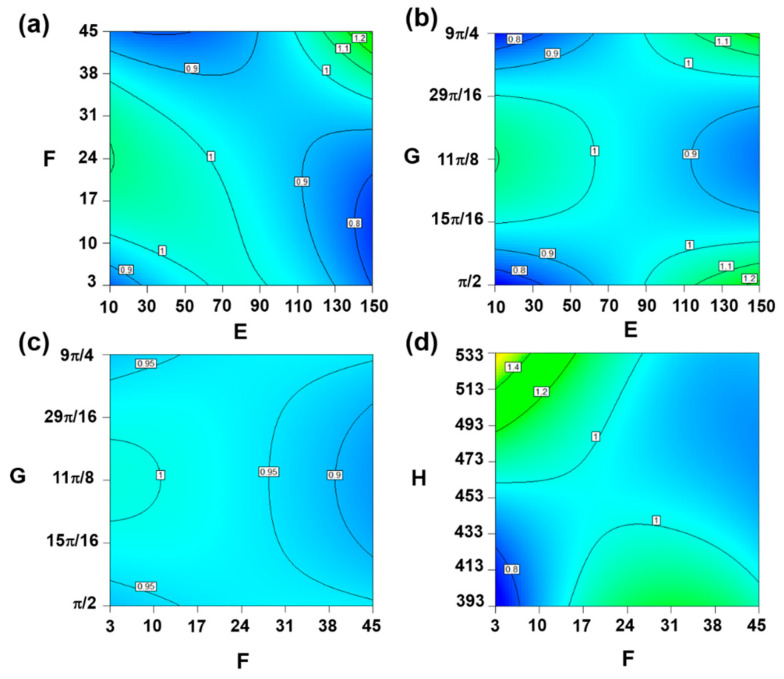
2D contour map of significant interaction items.

In Figure 7a, *S*_*re*_ was relatively higher when ambient amplitude *Z* and distribution density *k* varied around and lower than the central level, respectively. The same degree also occurred when the two variable both reached the maximum, showing an improving heat dissipation ability and the smaller effect on surroundings. The region with lower *S*_*re*_ will lead to the declining disorder and accelerate realizing steady state. *S*_*re*_ changed in a nonlinear way with the increase of both parameters and the effect of distribution density was more significant due to the higher gradient in its direction. The distribution of *S*_*re*_ in Figure 7b showed more symmetry properties. When ambient amplitude *Z* was lower than 90 K, a higher *S*_*re*_ was obtained if the value of phase *ϕ* near the central level was selected. For the rest range of ambient amplitude Z. However, it was necessary to avoid the middle value of phase *ϕ* in the given range and choose the extreme value. *S*_*re*_ still changed nonlinearly in the two directions, but with a similar changing gradient. In Figure 7c, the whole distribution of *S*_*re*_ and corresponding gradient in two directions were more uniform except the combination of lowest distribution density *k* and phase *ϕ* of middle value brough a slightly higher *S*_*re*_. We can consider that the interaction of these two factors has a relatively smaller influence on the thermodynamic properties of the cloaking system, which is also reflected by the fitting equation since the coefficient of this interaction item is much smaller than others. While in Figure 7d the uneven distribution of *S*_*re*_ obviously intensified, *S*_*re*_ increased both with the increase of distribution density and average hot end temperature. The gradient in both directions apparently increased and the best heat dissipation performance appeared when distribution density *k* was the lowest and average hot end temperature *T*_ave_ was the highest. The local area where the two variables reached the lowest value simultaneously in the given range can be regarded as an inefficient cloaking performance region. Above all, although the thermal cloak was applicable in a comprehensive environment, the specific application scope varied in different environmental conditions. These results could provide guidance for the further utilization and development of the thermal cloak in practical engineering.

## 4. Conclusions

In summary, we constructed a 2D cloaking structure combined with a dynamic external temperature field in the sinusoidal form with ambient amplitude, distribution density, phase, and temperature difference as variables. Thermodynamic indexes, namely local entropy production rate and response entropy, were selected to evaluate cloaking performance and environmental response. From the results and discussion above, we can draw the following conclusions:

1. The annulus cloaking structure performed well with infinitesimal local entropy production rate in the protected part and basically consistent characteristic distribution in the background compared to the bare plate. In the functional region, the difference of thermal conductivity between adjacent layers led to an alternating distribution of local entropy production rate, as well as the layer integration, which contributed the most heat dissipation and irreversible energy loss of the system.
2. From the area integral of local entropy production rate between layers, and of the cloaking region, the heat dissipation ability of thermal cloak increased with the both ambient amplitude and temperature difference. The effect of distribution density was relatively less obvious and the area integral of local entropy production rate of the cloaking region first decreased, then increased with the increase of phase. According to the change of response entropy, which is the integration of entropy variation between the cloaking region and its surroundings, it can be found that thermal cloak had corresponding application scopes for different environment variables.
3. Further, different schemes were conducted to obtain the fitting equation and analyze the interaction effect among different environmental variables, the rationality and significance of which were illustrated by the ANOVA method. Observed from the related 2D contour map, the appropriate interval of ambient amplitude, distribution density, phase, and temperature difference to apply a thermal cloak under the environment of a sinusoidal form can be intuitively evaluated.

The current work is an in-depth extension of the previous work [29], while our methods and results are beneficial to the novel use of the concept of entropy and valuable for further improving the working efficiency as well as exploring the potential engineering applications of the thermal cloak.

## Figures and Tables

**Table 1:** Changing range and levels of environmental variables.

Symbol	Variable		Levels	
		−1	0	1
E	ambient amplitude *Z* (K)	10	80	150
F	distribution density *k*	3	24	45
G	phase *ϕ*	π/2	11π/8	9π/4
H	average hot end *T*_ave_ (K)	393	463	533

**Table 2:** Modified cubic model results of ANOVA.

Source	Sum of Squares	df	Mean Square	F Value	*p* Value Prob > F	
model	1.70	14	0.12	19.12	<0.0001	significant
E	0.076	1	0.076	11.94	0.0039	significant
F	0.021	1	0.021	3.32	0.0901	significant
H	0.010	1	0.010	1.64	0.2217	
EF	0.067	1	0.067	10.48	0.0060	significant
EH	0.350	1	0.350	54.51	<0.0001	significant
FH	0.280	1	0.280	43.41	<0.0001	significant
F^2^	0.006	1	0.006	0.91	0.3564	
H^2^	0.100	1	0.100	15.77	0.0014	significant
E^2^F	0.084	1	0.084	13.15	0.0028	significant
E^2^H	0.170	1	0.170	27.13	0.0001	significant
EF^2^	0.130	1	0.130	20.88	0.0004	significant
EG^2^	0.320	1	0.320	50.24	<0.0001	significant
F^2^H	0.120	1	0.120	18.98	0.0007	significant
FG^2^	0.018	1	0.018	2.78	0.1176	
Residual	0.089	14	0.006			
Lack of Fit	0.089	10	0.009			
Pure Error	0.000	4	0.000			
Cor Total	1.79	28				
R-Squared	0.9503					
Adj R-Squared	0.9006					
Adeq Precison	18.981					
